# Levels of Heavy Metals in Grapevine Soil and Leaf Samples in Response to Seasonal Change and Farming Practice in the Cape Winelands

**DOI:** 10.3390/toxics11020193

**Published:** 2023-02-19

**Authors:** Amanda Mahlungulu, Learnmore Kambizi, Enoch Akinbiyi Akinpelu, Felix Nchu

**Affiliations:** Department of Horticultural Sciences, Faculty of Applied Sciences, Cape Peninsula University of Technology, P.O. Box 1906, Bellville 7535, South Africa

**Keywords:** seasonal variations in heavy metals, plant health, ICP-MS, crop cultivation

## Abstract

Heavy metal toxicity is a major threat to the health of both humans and ecosystems. Toxic levels of heavy metals in food crops, such as grapes, can have devastating effects on plant health and the market value of the produce. Two important factors that may influence the prevalence of heavy metals in grapevines are seasonal change and farming practices. The objectives of this study were (i) to conduct a detailed pioneer screening of heavy metal levels in soils and grapevine leaf tissues in selected wine farms and (ii) to study the influence of season and farming on heavy metal levels in soils and grapevine leaf tissues. Soil and grapevine leaf samples were collected from demarcated areas in selected vineyards in the Cape Winelands region of South Africa. The sampling was conducted in winter and summer from the same sites. The soil and leaf samples were analysed using inductively coupled plasma mass spectrometry (ICP-MS) techniques. The pooled data from the farms practising conventional or organic farming showed that seasonal variation had no significant effect (DF = 1, 22; *p* > 0.05) on the heavy metal contents in the soil. When the soil data from the winter and summer months were compared separately or pooled, the influence of agricultural practice was well-pronounced in As (DF = 1, 22, or 46; *p* < 0.05) and Cu (DF = 1, 22, or 46; *p* <0.05). The agricultural practice greatly influenced (DF = 1, 22; *p*< 0.05) Cu, As, Cr, and Hg uptake, with little effect on Ni, Co, Cd, and Hg leaf contents. Generally, the heavy metals studied (Cr, Co, Ni, Zn, As, Cd, Hg, and Pb) were substantially below the maximum permitted levels in plant and soil samples, per the recommendations of the WHO and E*_r_* indices, respectively. However, moderate contamination of the soils was recorded for Cr, Ni, Zn, and Pb. Remarkably, the Cu levels in the organic vineyard soils were significantly higher than in the conventional vineyards. Furthermore, based on the I_geo_ index, Cu occurred at moderate to heavy contamination levels.

## 1. Introduction

Natural and anthropogenic activities are responsible for the build-up of dangerous levels of heavy metals. Many factors affect the prevalence of heavy metals in grapevines [1]. According to Alagic et al. [2], one of the primary sources of heavy metals, such as lead, chromium, arsenic, zinc, cadmium, copper, and nickel, in soils is agricultural practices. The emissions of heavy metals from rapidly expanding industrial areas, mining tails, leaded gasoline and paint, fertilizers, animal manure, wastewater irrigation, and pesticides may contaminate the soil [3,4,5,6], leading to many environmental problems. Heavy metal toxicity is a major threat to the health of both humans and ecosystems; their accumulation in food crops, including grapes, can have devastating effects on plant health and the market value of the produce [2,7]. Briffa et al. [8] reviewed the toxicological effects of heavy metals on humans, including oxidative stress, liver damage, fever, pneumonia, asthma, brain damage, death, and DNA damage. 

Soils contaminated with heavy metals have become one of the major environmental problems around the world [9]. Industrial expansion, mine tailing, the combustion of fossil fuels, the spillage of petrochemicals, the disposal of high metal waste (e.g., batteries and metal scraps), atmospheric deposition, and agricultural practices may be the sources of heavy metals [5,6,10,11]. The agroecosystems are exposed to pollutants in fertilizers, biosolids, pesticides, and wastewater. Some farmers mix soil and sewage sludge, which may contain heavy metals [8]. A recent study on the level of atmospheric concentrations of commonly used pesticides successfully quantified carbaryl, chlorpyrifos, terbuthylazine, s-metolachlor, diazinon, tebuconazole, atrazine, simazine, malathion, and metazachlor in three agricultural regions (Grabouw, Hex River Valley and Piketberg) of the Western Cape, South Africa, and the concentrations were generally higher in the summer and during the spraying season [12]. Commonly used fungicides in vineyards, such as the Bordeaux mixture (Ca(OH)_2_ + CuSO_4_) and Mancozeb (C_4_H_6_MnN_2_S_4_)-based products, are important sources of Cu and Zn contamination, respectively. Phosphate fertilizers often contain Cd, Hg, and Pb impurities [13]. Agricultural soils may accumulate high levels of heavy metals, which has dire consequences for the quality and health of plants [14]. While it is helpful to regularly monitor the levels of heavy metals in agricultural soils, it is even more crucial to study the drivers of heavy metals in soils to achieve efficient and durable management of heavy metals. 

Mondol et al. [10] determined that environmental changes contribute to the differences in heavy metal uptake from soils. They also concluded that trace elements were higher during the dry season compared to the wet season [15]. Ullah et al. [16] suggest that this might result from lower pollution levels during the wet season as heavy rainfalls flush pollution into canals. A study by Oluyemi et al. [17] at a landfill in Nigeria showed that heavy metals were higher in the dry season than in the wet season. These claims are backed up by Osobamiro and Adewuyi [18], who studied three farm settlements in Ogun-State Southwest, Nigeria and found that heavy metal concentrations were higher in the dry season than during the wet season. The study suggests that high precipitation, leaching, erosion, and plant uptake may account for the reduction in heavy metal levels in the rainy season observed in the results of heavy metals from the three farm settlements.

Land-use patterns, including agricultural practices, profoundly influence soil quality, directly impacting heavy metal accumulation in the soil [19,20,21]. Many wine producers have adopted three farming practices to maximise production: conventional farming, polyculture, and organic farming [22]. In organic wine farming, no pesticides are used. It is a holistic farming system that promotes healthy and productive biodiversity while improving soil health [23]. Polyculture farming is the cultivation of different crops in the same space at the same time [24]. This practice slows down the soil degradation processes while improving soil fertility. Conventional farming involves using synthetic chemical fertilizers, pesticides, herbicides, and other genetically modified organisms in crop production. Conventional farming is one of the primary sources of heavy metals entering the food chain and posing a risk to environmental health [25].

The Cape Winelands is among the most important agriculture-producing regions in South Africa. It contributes approximately ZAR 26,223 million to the annual GDP of South Africa [26]. It is a world-renowned wine-producing region; hence, it is of utmost importance to study the heavy metal occurrence in grapevines of the Cape Winelands. It is essential to understand how ecological factors, especially the season and farming practices, influence the prevalence and accumulation of heavy metals in grapevines. The Cape Winelands region is an excellent model for studying the ecological dynamics of heavy metals. The Cape Winelands include Stellenbosch, Franschhoek, Constantia, Paarl, and Worcester.

The objectives of this study were (i) to conduct a detailed pioneer screening of heavy metal levels in soils and grapevine leaf tissues in selected wine farms and (ii) to study the influence of season and farming on heavy metal levels in soils and grapevine leaf tissues. This study revealed that farming practices influenced heavy metal contamination, especially Cu—its levels in organic vineyard soils were significantly higher than in conventional vineyards. However, generally, eight of the nine heavy metals studied (Cr, Co, Ni, Zn, As, Cd, Hg, and Pb) were substantially below the maximum permitted levels in plant and soil samples.

## 2. Materials and Methods

### 2.1. Experimental Design

Soil samples and grapevine leaves were collected from demarcated areas in selected vineyards in the Cape Winelands region of South Africa. The sampling was conducted in the winter and summer from the same sites. A deliberate effort was made to ensure that vineyards with different cultivation practices (organic, conventional, and mixed cropping) were selected for this study.

### 2.2. Site Characteristics

**Figure 1:**
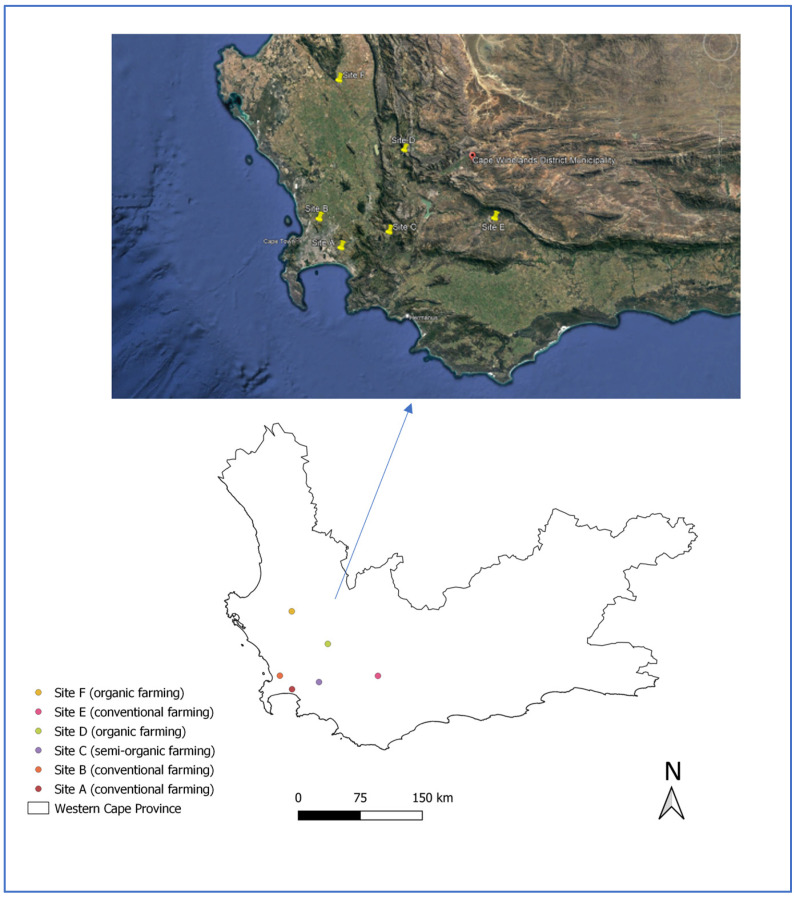
A map of the sampled vineyard sites in the Cape Winelands region; the map was created using QGIS and Google Earth software.

Six vineyards (sites) located in different regions of the Western Cape were selected for this study: Stellenbosch (A), Eikenbosch (B), Franschhoek (C), Wolseley (D), Robertson (E), and Piketberg (F) (Figure 1). Soils were obtained from vineyards with different cultivation approaches: organic (semi to 100% organic), conventional, and polyculture approaches.

### 2.3. Soil and Leaf Sampling

At each vineyard, four sampling points 200 m apart were randomly selected, and the sampling points were in the middle of the vineyard’s location for the points. From each sampling point, one kilogram of soil samples was collected after removing surface debris using a garden spade at a depth of 15–20 cm. The soil samples were placed in separate paper bags. Fresh leaf material (100 g) from randomly selected plants at sampling points that were 200 m apart was placed in a paper bag. A total of 48 soil and 48 leaf samples were collected from 6 vineyards in the Western Cape, South Africa. The sampling sites were geo-referenced (Table 1). The collection of samples from the same sampling points was carried out in two seasons (summer and winter). The soil and the leaf samples were analysed at the ICP-MS & XRF Laboratory, Stellenbosch University. Inductively coupled plasma mass spectrometry (ICP-MS) is a powerful technique for elemental trace analysis and is recommended for ultra-trace metals due to its increased sensitivity [27,28,29,30].

### 2.4. Sample Preparation and Analysis

The samples were air-dried and sieved (2 mm sieve) before testing. The concentrations (units: µg kg^−1^ or mg kg^−1^) of the major, minor, and trace elements of (ICP-AES and ICP-MS) Cr, Co, Ni, Cu, Zn, As, Cd, Pb, and Hg combined were determined as described by Berg et al. [31] with slight modifications. Portions of about 0.5 g (dry weight of plant samples) and 0.1 g (soil samples) were digested with 8 mL nitric oxide at 150 °C for 6–8 h. After cooling to room temperature, the samples were filtered, and demineralized water was added to a total volume of 50 mL. Calibration standards for the ICP-MS analysis were prepared from multi-element stock solutions (Spec-troscan, Teknolab As, N-1440 Drsbak). The ICP-MS instrument was calibrated with standard solutions of 50 and 250 ng mL^−1^. For the major elements, an additional standard of 1000 ng mL^−1^ was used. All the calibration standards and blanks were matched with the nitric acid concentration of the samples. The certified reference material 1573a (tomato leaves) was used to validate the analytical methods for determining the botanical materials’ major, minor, and trace elements. Accuracy and precision for the soil samples were achieved by using internal quality control standards (WQB-1). The result of the digested solution in mg/L obtained from the ICP was multiplied by the dilution factor in the digestion process using the following formula: mg kg^−1^ = mg L^−1^ × [(Final volume mL)/(weight of sample g)]. Analyses were performed on a Plasma Quad I ICP- MS instrument. The ICP-MS was equipped with a peristaltic pump (Ismatec Reglo 100) and a Meinhard nebulizer. The permissible limits for heavy metals in edible plants that were published by the World Health Organization [32,33] and the Food and Agriculture Organization of the United Nations (FAO) were used as standards for the comparison and classification of heavy metal levels into three categories (low, optimum, and high); the levels for the individual heavy metals are as follows: 0.5 µg g^−1^ arsenic (As), 0.02 µg g^−1^ cadmium (Cd), 1.3 µg g^−1^ chromium (Cr), 0.01 µg g^−1^ cobalt (Co), 10 µg g^−1^ copper (Cu), and 0.03 µg g^−1^ mercury (Hg).

### 2.5. Contamination and Ecological Risk Assessment

Contamination indices were used to evaluate the influence of anthropogenic activities on the accumulation of heavy metals in the farms (geo-accumulation index [I_geo_]) and the ecological risks associated with heavy metal levels (contamination factor [C_f_] and ecological risks [E_r_]). The following formula was used [34,35]:

I_geo_ = log_2_[C*_n_*/1.5B*_n_*]

where C*_n_* is the measured concentration of metal in the soil and

B*_n_* is the background value of a metal. 

The background values (mg kg^−1^) for Cr (5.82), Cu (2.98), Cd (0.62), Zn (12), Hg (0.15), and Pb (2.99) were from South Africa [36], the value for As (20) was from the Netherlands [37], and the value for Co (18) was from China [38]. To compensate for possible variations in the background values and minor anthropogenic influences, a factor of 1.5 was used [34]. The degree of metal contamination in soils as defined by Muller [39], with seven soil quality levels ranging from 1 (uncontaminated) to 6 (extremely contaminated), was used (Table 2). 

The ecological risk index of each heavy metal was determined using the method developed by Hakanson [40] (Table 2). The following equations were used [34,40]:

C_f_ = C*_n_*/B*_n_*

E*_r_* = T*_r_* × C*_f_*

where T_r_ is the toxic response factor for each given pollutant,

C*_f_* is the contamination factor for each heavy metal,

C*_n_* is the measured level of each heavy metal in the sediment,

B*_n_* is the background level of each heavy metal, and

E*_r_* is the ecological risk index.

The toxic response factors [40] are: Cr (2), Co (5), Cu (5), Cd (30), Ni (5), Zn (1), As (10), Hg (40), and Pb (5).

### 2.6. Statistical Analysis

Heavy metal concentrations in the soils and leaf tissues obtained during the winter and summer months from each farm were compared using a one-way analysis of variance (ANOVA). The heavy metal concentrations in the soils and leaf tissues obtained from farms with different farming practices were compared using a one-way analysis of variance (ANOVA). SPSS was used to process and analyse the data.

## 3. Results

### 3.1. Heavy Metals in Soil Samples

#### 3.1.1. Levels of Heavy Metals in Soil Samples

Three of the farm sites (Sites A, B, and E) that were sampled practise conventional farming, and the other three farms practise organic farming (Sites D, E, and F). Meanwhile, four farm sites had polycultures, three of which were conventional farms. The average concentrations of heavy metals in the soil samples from six study sites in the Cape Winelands are given in Table 3. The mean concentration of heavy metal in the soil was highest for chromium (58.738 ± 2.988 mg kg^−1^), and the lowest was observed for Hg (0.015 ± 0.0002 mg kg^−1^) at site F. The mean concentrations of Cd and Hg in the soil samples are generally low across all the sites. 

#### 3.1.2. Effect of Seasonal Variation on Heavy Metal Deposits in the Soil

**Figure 2:**
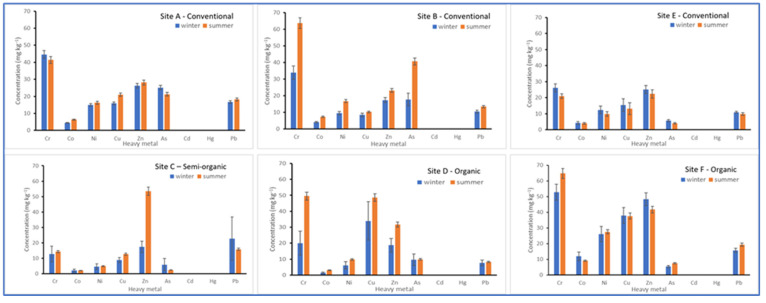
Seasonal fluctuation of heavy metals in soil samples from different grapevine sites (**A**–**F**) in the Cape Winelands.

The seasonal variations in the distribution of some of the selected heavy metals in soil samples from the Cape Winelands are shown in Figure 2. The levels of Cd and Hg in all the vineyards are generally minimal. Site E recorded the lowest levels of heavy metals in the soil samples analysed. The heavy metal contents of the soil did not vary significantly (DF = 1, 6; *p* > 0.05) between the winter and summer for all the study sites. Furthermore, when the data from the farms that practice conventional or organic farming were pooled, the seasonal variation had no significant effect (DF = 1, 22; *p* > 0.05) on the heavy metal contents in the soil.

#### 3.1.3. Effect of Agricultural Practice on Heavy Metal Deposits in the Soil

**Figure 3:**
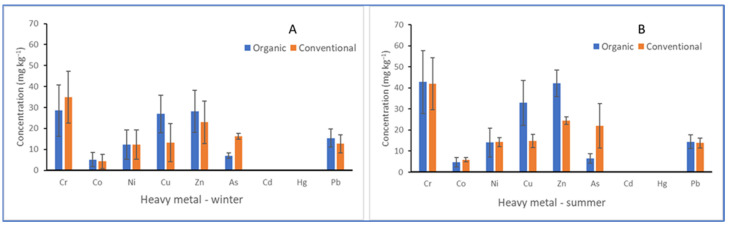
Influence of agricultural practices on heavy metal deposits in soils during the winter (**A**) and summer (**B**) in vineyards in the Cape Winelands.

The impact of agricultural practices (conventional; Sites A, B, and E) and organic practices (sites C, D and F) on heavy metal deposits in the soil is shown in Figure 3. When the soil data from the winter and summer months were compared separately or pooled, the influence of agricultural practice was well-pronounced in As (DF = 1, 22, or 46; *p* < 0.05) and Cu (DF = 1, 22, or 46; *p* < 0.05). There were no significant differences in the overall heavy metal deposits in the soil between organic and conventional agricultural practices in both the summer (DF = 1, 16; F = 0.09; *p* = 0.76) and winter (DF = 1, 16; F = 0.02; F = 0.76). The ecological risk index based on the contamination factors and background levels showed low ecological risk in the vineyards for eight of the nine heavy metals assessed—the E_r_ was below 40, corresponding to low risk (Table 2 and Table 4). Meanwhile, the geo-accumulation index (E*_r_* ˂ 0) indicated a low level of soil contamination for Co, As, Cd, and Hg (Table 4), and neither season nor farming practice had a significant effect on soil contamination. However, moderate contamination of the soils was recorded for Cr, Ni, Zn, and Pb (Table 4). The Cu I_geo_ (2.329 ± 0.674 − 2.669 ± 0.597) and E*_r_* (45.068 ± 15.234 − 55.248 ± 17.883) values in the organic farms were relatively higher than the Cu I_geo_ (1.512 ± 0.297 − 1.661 ± 0.303) and E*_r_* (22.249 ± 4.043 − 24.820 ± 5.381) in the conventional farms, suggesting moderate to heavy levels of geochemical contamination and moderate ecological risk (Table 4). 

### 3.2. Heavy Metals in Plant Samples

#### 3.2.1. Levels of Heavy Metals in Plant Samples

The average concentrations of heavy metals in the plant samples from the six study sites in the Cape Winelands are provided in Table 5. The highest mean concentration of heavy metals in the plant samples was observed for Cu (87.098 ± 19.481 mg/kg) at site D, and the lowest was observed for Cd (0.002 ± 0.0004 mg/kg), also at site D. There were significant (DF = 5, 18; *p* < 0.05) variations in the heavy metal contents (Cr, Cu, As, Cd, Hg, and Pb) in the plant leaves among the sites. 

#### 3.2.2. Effect of Agricultural Practice on Heavy Metal Uptake by Plant Samples

**Figure 4:**
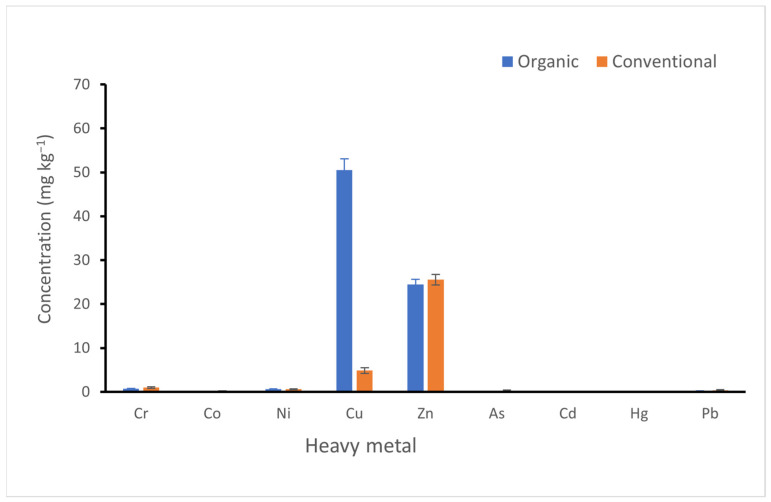
Influence of agricultural practices on heavy metal uptake by plant samples from vineyards of the Cape Winelands.

Leaf samples from eight cultivars of grapevine plants occurring in the farms were analysed. To determine the impact of agricultural practices on heavy metals, pooled data from conventional (A, B and E) and organic (sites C, D and F) farming sites were statistically compared (Figure 4 and Table 5). The agricultural practice significantly influenced (DF = 1, 22; *p* < 0.05) Cu, As, Cr, and Hg uptake, with little effect on Ni, Co, Cd, and Hg. Generally, the heavy metals were substantially below the maximum permitted levels in plants. 

## 4. Discussion

A key finding of this study is that the heavy metal contents in soils and grape leaves are below the maximum allowed concentrations of heavy metals in the leaf samples, based on the recommendations of the WHO [33]. Furthermore, the heavy metal concentrations in the soil for eight of the nine heavy metals posed low ecological risk based on the classification of ecological risk heavy metal pollution (40). This is good news for wine consumers and the wine industry in South Africa as the Cape Winelands is the largest wine-producing region on the African continent [41,42]. In addition, the seasonal change did not significantly influence variations in the heavy metals. However, farming practices influenced the accumulations of As and Cu, suggesting that pesticide application is a more important factor influencing heavy metal contents in the Cape Winelands. Cu contamination levels in organic farm soils had higher I_geo_ values (2.3–2.7), which corresponded to moderately to heavily contaminated soils compared with those in conventional farms. In addition to the over-dependence on agrochemicals, rapid industrialization and urbanization contribute significantly to heavy metal contamination through the high use of metal, leaded gasoline, paint, and petrochemical waste disposal and atmospheric deposition [43,44].

Cu and As varied significantly between the farms that employed organic and conventional farming practices. These two elements are contained in some well-known pesticides used in the cultivation of grapevines [45]. The levels of As were higher in the farms that practice conventional farming. This was expected because many insecticides used to control pests in grapevines have arsenic compounds. The application of foliar fungicides in vineyards and orchards can increase the soil concentration of heavy metals, such as copper (Cu) and zinc (Zn), up to the toxicity threshold for fruit trees and cover crops [13]. However, remarkably, the Cu concentrations in the organic vineyards were higher than in the conventional vineyards in the current study. The Cu I_geo_ and E_r_ values in the organic farms were higher relative to the conventional farms and corresponded to moderate to heavy contamination and moderate ecological risk, respectively. Vannini et al. [35] also reported similar findings in agricultural soils of the Valdichiana area, Tuscany, Italy; the C_f_ and I_geo_ indices for Cu were higher than for the other heavy metals, and they attributed those findings to the increased use of Cu-based products. The accumulation of Cu in soil and plant tissues could be influenced by many factors other than pesticides, such as the mineralization of organic matter, microorganisms, and minerals in the rock. It is worth noting that organic amendments such as compost and manure, which are widely used in organic farming, bind with Cu more tightly than other micronutrients [46]. Previous studies have investigated the levels of heavy metals in grapefruits in Spain and China [47,48]. 

This study showed that the season did not affect the heavy metal levels. The results from previous studies suggest that heavy metal concentrations in soils, rivers, and leaves vary with the season; generally, higher heavy metal concentrations are more prevalent in the dry season than in the rainy season [17,49]. In a study by Okoro et al. [50] on the concentrations of heavy metals in seawater from Cape Town harbour, South Africa, the authors reported that Sn and Cd occurred at higher levels in the summer, while Hg, Pb, and As were more prevalent in the winter. It is worth noting that the Cape Peninsula region has a Mediterranean climate, characterised by hot and dry summers and cold and rainy winters [51].

Although this study only investigated the concentrations of heavy metals in vineyard soils and grapevine leaves, the results are very relevant because the use of Cu- and Zn-based pesticides in vineyards can increase the levels of these metals in wines and grapes. In the current study, the geochemical analysis showed that in addition to Cu, the heavy metals Ni, Zn, Cr, and Pb showed moderate soil contamination. In a study conducted in Sri Lanka, Prabaga et al. [11] found that most of the accumulated metals are mainly concentrated in the leaves of the grape tree than in the fruit. A survey carried out on the west coast of the Oristano province (Sardinia, Italy) revealed that cobalt occurred at a greater level than the legal limit in one vineyard, and the long-term use of copper-based fungicides in vineyards does not represent a cause of concern for the studied areas [52]. A study that investigated cadmium, copper, lead, and zinc concentrations in wines and alcohol-containing drinks from Italy, Bulgaria, and Poland revealed that these metals occurred in low concentrations; however, the Cu and Zn concentrations were highest in the Italian wines (Cu = 0.13 ± 0.05 mg L^−1^; Zn = 0.83 ± 0.56 mg L^−1^) and lowest in the Polish products (Cu = 0.04 ± 0.001 mg L^−1^; Zn = 0.18 ± 0.16 mg L^−1^) [53]. 

## 5. Conclusions

Four (Co, As, Cd, and Hg) of the nine heavy metals occurred at very low concentrations in the vineyard soils and posed low contamination and ecological risks. However, moderate contamination of the soils was recorded for Cr, Ni, Zn, and Pb. Notably, the Cu levels in the organic vineyard soils were significantly higher than in the conventional vineyards, which is surprising and requires further investigation because Cu-based pesticides are generally not used in organic farming. The season had no significant influence on heavy metal contamination. This study provides comprehensive baseline data on heavy metals in vineyard soils and grapevine leaves in the Cape Winelands. The findings of this study can be applied when adopting farming practices that promote a reduction in metals and also highlight the need for continuous monitoring of toxic metals, even in organic farming, for healthier agroecosystems. 

## Figures and Tables

**Table 1:** The coordinates of sampled vineyards in the Cape Winelands, the location, the sampled grapevine cultivars, and the farming practices.

Coordinates	Site	Town	Grapevine Cultivars Sampled	Farming Practice
Y = −34.0170461 X = 18.7550072	A *	Stellenbosch	Cabernet sauvignon and Cabernet franc	Conventional
Y = −33.8347509 X = 18.591131	B *	Eikenbosch	Sauvignon blanc and Cabernet franc	Conventional
Y = −33.9205238 X = 19.1186237	C *	Franschhoek	Merlot and Cabernet sauvignon	Sem-organic
Y = −33.4056598 X = 19.2374146	D	Wolseley	Shiraz, Sèmillon, Merlot, and Sauvignon blanc	Organic (certified)
Y = −33.836914 X = 19.9131483	E *	Robertson	Chardonnay, Sauvignon, and Sauvignon blanc	Conventional
Y = −32.96663 X = 18.75134	F	Piketberg	Cabernet sauvignon, Cabernet sauvignon, Merlot, and Shiraz	Organic (certified)

* Evidence of polyculture farming observed.

**Table 2:** Classes of metal contamination, I_geo_ [39], and the ecological risk for metal pollution, E_r_, [40].

I_geo_ Class	I_geo_ Value	Soil Quality Based on I_geo_ Value	E*_r_*	Ecological Risk of Single Metal
0	<0	Uncontaminated	E*_r_* ˂ 40	Low risk
1	0–1	Uncontaminated to moderately contaminated	40 ≤ E*_r_* ˂ 80	Moderate risk
2	1–2	Moderately contaminated	80 ≤ E_r_ ˂ 160	Considerable risk
3	2–3	Moderately contaminated to heavily contaminated	160 ≤ E*_r_* ˂ 320	High risk
4	3–4	Heavily contaminated	E*_r_* ≥ 320	Very high risk
5	4–5	Heavily to extremely contaminated		
6	>5	Extremely contaminated		

**Table 3:** Average concentrations of selected heavy metals in soil samples from different sites collected in summer and winter.

Sites	Heavy Metal Concentrations (SEM) mg kg^−1^ in Soils									
	* FP	Cr	Co	Ni	Cu	Zn	As	Cd	Hg	Pb
A ***	C	42.933 ± 1.622	5.399 ± 0.964	15.568 ± 0.654	18.471 ± 2.508	27.171 ± 0.913	23.177 ± 1.917	0.019 ± 0.002	0.042 ± 0.0005	17.488 ± 0.763
B ***	C	48.849 ± 14.948	5.673 ± 1.592	13.155 ± 3.609	9.343 ± 0.891	20.271 ± 2.884	29.166 ± 11.442	0.024 ± 0.004	0.030 ± 0.011	11.929 ± 1.498
C ***	O^	13.505 ± 0.749	1.987 ± 0.001	4.695 ± 0.158	10.719 ± 1.876	35.406 ± 18.001	4.074 ± 1.752	0.044 ± 0.025	0.032 ± 0.0004	19.285 ± 3.452
D	O	34.763 ± 14.738	2.267 ± 0.835	7.931 ± 1.800	41.275 ± 7.365	25.167 ± 6.477	9.751 ± 0.126	0.027 ± 0.013	0.018 ± 0.010	7.896 ± 0.270
E ***	C	23.586 ± 2.578	4.129 ± 0.087	11.112 ± 1.281	14.266 ± 1.101	23.690 ± 1.353	4.900 ± 0.826	0.022 ± 0.0004	0.019 ± 0.003	10.376 ± 0.557
F	O	58.738 ± 2.988	10.550 ± 0.7047	26.812 ± 0.369	37.687 ± 0.071	44.980 ± 1.651	6.455 ± 0.515	0.032 ± 0.005	0.015 ± 0.0002	17.550 ± 1.821
** FAO/WHO-ML		100	50	50	100	50	20	3.0	-	100

* FP = Farming practice—Conventional(c)/Organic(o)/Semi-organic (^), SEM = Standard Error of Mean, ** ML = Maximum level permitted in soil by [33]; *** = sites which also practised polyculture; - = data not available

**Table 4:** Contamination factor (C_f_), ecological risk index (E*_r_*), and geo-accumulation Index (I_geo_) (Mean ± SE) of heavy metals occurring in the soils of the vineyards in the Cape Winelands.

Heavy Metal	Season	Farming Practice	C*_f_*	E*_r_*	I_geo_
	Winter	Conventional	5.992 ± 0.916	11.984 ± 1.832	1.964 ± 0.222
Cr		Organic	4.899 ± 2.114	9.798 ± 4.228	1.447 ± 0.604
	Summer	Conventional	7.223 ± 2.123	14.446 ± 4.246	2.126 ± 0.466
		Organic	7.358 ± 2.567	14.716 ± 5.135	2.034 ± 0.672
	Winter	Conventional	0.236 ± 0.006	1.179 ± 0.029	−2.670 ± 0.035
Co		Organic	0.285 ± 0.190	1.424 ± 0.951	−3.059 ± 0.952
		Conventional	0.327 ± 0.053	1.636 ± 0.267	−2.239 ± 0.256
	Summer	Organic	0.264 ± 0.124	1.319 ± 0.618	−2.815 ± 0.654
	Winter	Conventional	3.582 ± 0.452	17.908 ± 2.261	1.232 ± 0.187
Ni		Organic	3.571 ± 2.020	17.853 ± 10.100	0.804 ± 0.779
	Summer	Conventional	4.161 ± 0.649	20.805 ± 3.245	1.432 ± 0.249
		Organic	4.095 ± 2.011	20.475 ± 10.055	1.085 ± 0.728
	Winter	Conventional	4.449 ± 0.809	22.249 ± 4.043	1.512 ± 0.297
Cu		Organic	9.014 ± 3.047	45.068 ± 15.234	2.329 ± 0.674
	Summer	Conventional	4.964 ± 1.076	24.820 ± 5.381	1.661 ± 0.303
		Organic	11.049 ± 3.576	55.248 ± 17.883	2.669 ± 0.597
	Winter	Conventional	1.908 ± 0.231	1.908 ± 0.231	0.324 ± 0.188
Zn		Organic	2.344 ± 0.840	2.344 ± 0.840	0.476 ± 0.474
	Summer	Conventional	2.044 ± 0.149	2.044 ± 0.149	0.439 ± 0.102
		Organic	3.520 ± 0.524	3.520 ± 0.524	1.198 ± 0.218
	Winter	Conventional	0.809 ± 0.282	8.091 ± 2.822	−1.135 ± 0.643
As		Organic	0.348 ± 0.067	3.479 ± 0.669	−2.157 ± 0.260
	Summer	Conventional	1.099 ± 0.528	10.990 ± 5.276	−0.980 ± 0.988
		Organic	0.328 ± 0.111	3.281 ± 1.115	−2.432 ± 0.640
	Winter	Conventional	0.036 ± 0.004	1.097 ± 0.134	−5.379 ± 0.173
Cd		Organic	0.037 ± 0.011	1.121 ± 0.339	−5.451 ± 0.414
	Summer	Conventional	0.034 ± 0.001	1.029 ± 0.042	−5.453 ± 0.059
		Organic	0.073 ± 0.020	2.179 ± 0.617	−4.483 ± 0.407
	Winter	Conventional	0.186 ± 0.049	7.436 ± 1.961	−3.105 ± 0.357
Hg		Organic	0.122 ± 0.046	4.865 ± 1.837	−3.844 ± 0.572
	Summer	Conventional	0.221 ± 0.057	8.834 ± 2.267	−2.890 ± 0.456
		Organic	0.168 ± 0.035	6.720 ± 1.387	−3.232 ± 0.339
	Winter	Conventional	4.242 ± 0.671	21.209 ± 3.354	1.466 ± 0.215
Pb		Organic	5.139 ± 1.460	25.693 ± 7.301	1.639 ± 0.463
	Summer	Conventional	4.622 ± 0.813	23.110 ± 4.065	1.578 ± 0.257
		Organic	4.835 ± 1.106	24.176 ± 5.530	1.598 ± 0.376

**Table 5:** Average concentrations of selected heavy metals in grapevine leaf samples from different sites (vineyards) in the Cape Winelands.

Sites	Heavy Metal Concentration (SEM) mg kg^−1^									
	* FP	Cr	Co	Ni	Cu	Zn	As	Cd	Hg	Pb
A ***	C	0.959 ± 0.057 ab	0.240 ± 0.053 a	0.566 ± 0.049 a	4.230 ± 0.328 a	27.906 ± 2.230 ab	0.318 ± 0.046 ab	0.007 ± 0.001 ab	0.017 ± 0.002 ab	0.373 ± 0.045 ab
B ***	C	1.335 ± 0.164 a	0.269 ± 0.047 a	0.574 ± 0.047 a	4.256 ± 0.458 a	23.987 ± 3.138 ab	0.454 ± 0.102 a	0.008 ± 0.0008 ab	0.017 ± 0.001 ab	0.619 ± 0.057 a
C ***	O^	0.620 ± 0.081 b	0.107 ± 0.011 a	0.431 ± 0.058 a	3.957 ± 0.364 a	32.289 ± 5.858 a	0.119 ± 0.030 b	0.018 ± 0.005 a	0.018 ± 0.002 ab	0.307 ± 0.063 b
D	O	0.572 ± 0.063 b	0.103 ± 0.018 a	0.461 ± 0.069 a	87.098 ± 19.481 b	24.192 ± 2.730 b	0.125 ± 0.022 b	0.002 ± 0.0004 b	0.020 ± 0.0005 ab	0.295 ± 0.083 b
E ***	C	0.699 ± 0.069 b	0.200 ± 0.044 a	0.821 ± 0.203 a	6.082 ± 0.885 a	24.789 ± 1.437 ab	0.106 ± 0.009 b	0.016 ± 0.006 ab	0.014 ± 0.002 a	0.165 ± 0.035 b
F	O	0.973 ± 0.131 ab	0.298 ± 0.106 aa	1.104 ± 0.372 a	60.603 ± 7.971 bc	16.848 ± 1.937 ab	0.117 ± 0.021 b	0.004 ± 0.001 ab	0.023 ± 0.003 b	0.197 ± 0.034 b
** FAO/WHO-ML		1.3	50	10	10	99.4	0.0005	0.02	0.1	2

* FP = Farming practice—Conventional(c)/Organic(o)/Semi-organic (^), SEM = Standard Error of Mean, ** ML = Maximum level permitted in edible plants by [33]; *** = sites with evidence of polyculture farming; means with the same lowercase letters (a or b or c) are not significantly different.

## Data Availability

The data presented in this study will be openly available in Figshare at DOI: 10.25381/cput.21821703.
